# The Effects of Hypoxia on the Immune-Modulatory Properties of Bone Marrow-Derived Mesenchymal Stromal Cells

**DOI:** 10.1155/2019/2509606

**Published:** 2019-10-08

**Authors:** Zsolt Fábián

**Affiliations:** ^1^Department of Medicinal Chemistry, Molecular Biology and Pathobiochemistry, Semmelweis University, Budapest, Hungary; ^2^School of Medicine, Faculty of Clinical and Biomedical Sciences, University of Central Lancashire, Preston, UK

## Abstract

The therapeutic repertoire for life-threatening inflammatory conditions like sepsis, graft-versus-host reactions, or colitis is very limited in current clinical practice and, together with chronic ones, like the osteoarthritis, presents growing economic burden in developed countries. This urges the development of more efficient therapeutic modalities like the mesenchymal stem cell-based approaches. Despite the encouraging *in vivo* data, however, clinical trials delivered ambiguous results. Since one of the typical features of inflamed tissues is decreased oxygenation, the success of cellular therapy in inflammatory pathologies seems to be affected by the impact of oxygen depletion on transplanted cells. Here, we examine our current knowledge on the effect of hypoxia on the physiology of bone marrow-derived mesenchymal stromal cells, one of the most popular tools of practical cellular therapy, in the context of their immune-modulatory capacity.

## 1. Introduction

Mesenchymal stromal cells (MSCs) are considered to be a promising tool for cellular therapy in various human pathologies. These include both chronic and acute inflammatory conditions like, for instance, osteoarthritis, rheumatoid arthritis, colitis, septic conditions, or graft-versus-host disease. Despite numerous studies indicating the efficacy of MSCs in inflammatory animal models, clinical trials reported controversial outcomes. Behind the diverse pathogenesis of the distinct inflammatory conditions, local hypoxia is considered to be a common pathogenic factor. Indeed, inflammation is often accompanied by metabolic hypoxia in various inflammatory diseases. Bone marrow-derived MSCs (BMSCs) naturally reside in a severely oxygen-depleted microenvironment that supports the concept of their use in the cellular therapy of inflammatory conditions [1, 2]. Since differential oxygen levels exert complex effects on cellular physiology, here, we review our current understanding on the interplay between the immune-modulatory effects and hypoxic response of BMSCs and formulate problems to be addressed in order to develop more efficient BMSC-based medical applications for inflammatory pathologies.

## 2. Bone Marrow-Derived Mesenchymal Stromal Cells

BMSCs, similar to mesenchymal stem cells isolated from other tissues, are multipotent cells that possess the plasticity to differentiate into various cell types of mesenchymal origin [3, 4]. It is noteworthy, however, that some studies on BMSC plasticity widened the range of tissues BMSCs which could be potentially differentiated further [5–9]. These data suggest the existence of trans-lineage plasticity in BMSC populations and raise the question if BMSCs, or at least a subset of these cells, are rather pluripotent. Independent of this classification/semantical uncertainty, their plasticity fueled the idea that they have great medical potential in pathologies affecting tissues with poor regenerative capacity like the cartilage, myocardium, or tendons [10]. In support of this concept, intra-articular administration of BMSCs to patients suffering from knee cartilage damage was reported beneficial based on clinical scorings, though the fate of transplanted cells remained unevaluated [11]. Another study found that the use of hyaluronic acid augments the effects of transplanted BMSCs indicating that the importance of the surrounding microenvironment in the efficacy of the BMSC-based cellular therapy [12]. In contrast, however, no statistically significant improvement was reported in osteoarthritis patients after cellular therapy with BMSCs differentiated toward chondrogenic lineages prior transplantation raising the question if efficacy observed in trials was mediated by direct cartilage repair [13]. Indeed, tissue damage is often accompanied by inflammation so one can speculate that for successful tissue regeneration, transplanted cells have to, ideally, modulate the inflammatory milieu. Clinical reports on the efficient use of hBMSCs in high-risk pediatric acute leukemia patients to improve platelet and neutrophil recovery, apparently, support this hypothesis. Although data are not consistent among published clinical trials, BMSCs were considered to be responsible for the apparent attenuation of the graft-versus-host reactions, possibly, through their anti-inflammatory effects posttransplantation [14, 15]. An independent phase I/II study, however, reported that the majority of the patients either showed partial response or did not respond to BMSC-based cellular therapy at all [16].

Similarly, conflicting results have been published in relation to other inflammatory conditions as well. *In vivo* studies on the potential use of BMSCs in inflammatory conditions of the respiratory system showed promising results. In rodent smoke-induced lung damage models, rat BMSCs (rBMSCs), administered via the trachea, repressed the expression of proinflammatory cytokines tumour necrosis alpha (TNF-*α*), interleukin-1 beta (IL-1*β*), interleukin-6 (IL-6), and the monocyte chemoattractant protein 1 (MCP-1) in the lung parenchyma. Parallel, induction of the vascular endothelial growth factor (VEGF), its type 1 receptor (VEGFR1), and the transforming growth factor beta (TGF-*β*) was reported in lung tissue homogenate suggesting an overall anti-inflammatory pulmonary effect of rBMSCs [17]. In a follow-up study, the same group reported repression of cyclooxygenase-2 (COX-2) and its downstream effector prostaglandin E2 (PGE2) production in alveolar macrophages as a possible mechanism behind the rBMSC-mediated anti-inflammatory pulmonary effects [18]. Independent studies in mouse lipopolysaccharide-induced pneumonia models also suggest that BMSCs may mediate the anti-inflammatory effects through the modulation of macrophage functions [19–21]. Despite the promising results in animal models, however, a multicenter phase II study in over 60 chronic obstructive pulmonary disease (COPD) patients did not find significant effects of the use of intravenous infusions of allogenic human BMSCs (hBMSCs) [22]. In accordance, only weak efficacy of the intravenous transplantation of hBMSCs was observed in a recent phase I trial with patients suffering from acute respiratory distress syndrome (ARDS) raising the question of both the mechanisms underlying the controversial responses and optimized protocols for improved therapeutic efficacy [23].

Despite the likely diverse extracellular milieu present in distinct inflammatory conditions, one could speculate that the determining factor of the BMSC-based cellular therapy outcome is the differential oxygen levels cells are exposed to before, during, and after transplantation. Indeed, CD4^+^ T cells, for instance, adapt successfully to hypoxic conditions and this adaptation is accompanied by elevated secretion of a cohort of proinflammatory cytokines including IL-1*β*, IL-6, IL-8, IL-10, and MCP-1 [24]. BMSCs are also naturally resistant to a severely oxygen-depleted environment, but cells that are transplanted, for instance, into joints have to exert their immune-modulatory functions in a fundamentally differentially oxygenated milieu compared to those trapped in the lungs after intravenous administration [25]. Thus, understanding the adaptation of BMSCs to various oxygen levels might be one of the keys for establishing better off-shelf BMSC products and more efficient BMSC-based therapeutic protocols for inflammatory diseases.

## 3. Hypoxia

Although hypoxia is typically associated with pathophysiologic states, it is, actually, present in physiologic conditions as well. Indeed, oxygen depletion occurs from the very first stages of embryogenesis and remains present during the whole morphogenesis. Local hypoxia not only is responsible for the proliferation of placental epithelial stem cells, the cytotrophoblasts, but also serves as an orientation signal for their invasion into the uterus, a critical factor of placental development [26]. Hypoxic tissues are present in the growing embryo elsewhere as well, and their common presence in various experimental model species including rodents and birds suggests that the phenomenon is a general property of the vertebrate embryogenesis [27]. Although its distribution shows a temporospatial variation, hypoxic regions remain detectable throughout the whole morphogenesis. In the 14.5E mouse embryo, for instance, extensive oxygen-depleted regions are present in the midbrain, pituitary gland, spine cord, vertebrae, and sternum as well as in tissues of the tongue, heart, lungs, and intestine [28]. *In vivo* data also showed that artificial modification of oxygen levels upon embryonic development leads to severe placental malformations or abnormal morphogenesis suggesting that the embryonic hypoxia cannot be exclusively considered a passive outcome of the massive expansion of embryonic tissues but rather a tightly regulated organogenetic signal [29, 30]. This also underlines the importance of the hypoxic milieu in the physiology of pluripotent cellular species responsible for tissue organogenesis. Extremely low oxygen tensions are also present in tissues under physiologic condition during the postembryonic life. This “physiologic” hypoxia is present even in well-vascularized organs like the heart, kidneys, or brain (Table 1.). Moreover, recent findings on the central role of the microbiome-mediated oxygen-depletion of the intestinal epithelium in the maintenance of the intestinal barrier function suggest that the physiologic role of hypoxia in adult tissues might be more critical than it has been, previously, anticipated [31].

### 3.1. The Molecular Machinery of Hypoxic Adaptation

**Figure 1:**
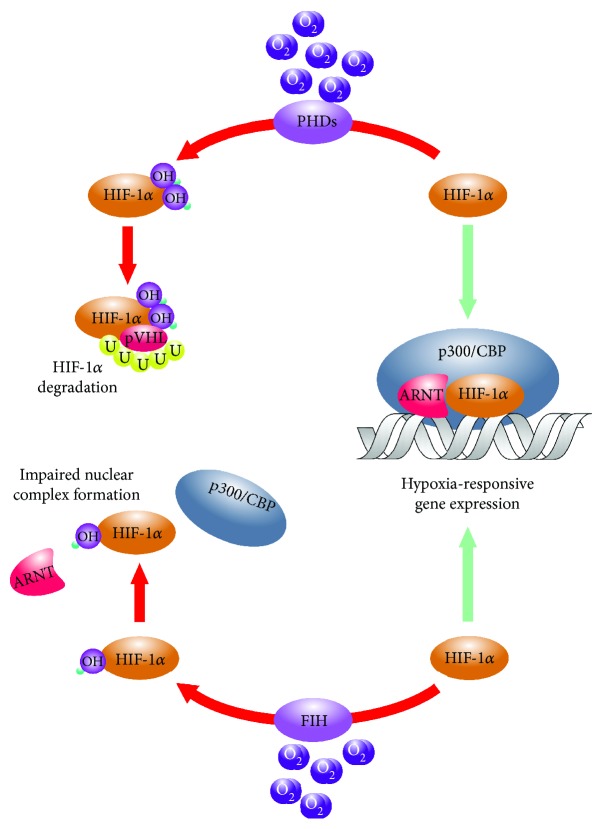
Hydroxylation-mediated regulation of the HIF-*α* subunits. The primary posttranslational regulation of the HIF-*α* polypeptides is mediated by the prolyl-4-hydroxlase-1, prolyl-4-hydroxlase-2, and prolyl-4-hydroxlase-3 (PHDs) that catalyze the hydroxylation of conserved proline residues. This leads to the ubiquitylation and subsequent proteasomal degradation of the HIF-*α* subunits in the presence of oxygen. A complimentary hydroxylation catalyzed by the asparagine hydroxylase termed factor inhibiting HIF (FIH) that prevents the association of HIFs with their transcriptional coactivator p300.

Independent of the nature of hypoxia, metazoan cells need to adapt to the oxygen-depleted milieu to ensure the balance of their oxygen-dependent metabolic homeostasis and survival. Adjustment of cellular metabolism in hypoxia is, primarily, orchestrated by helix-loop-helix type transcription factors termed hypoxia-inducible factors (HIF) [32] (Figure 1). The heterodimer HIFs, besides the shared beta one, consist of distinct alpha subunits that are steadily degraded by the 26S proteasome system in oxygenated cells [33]. This “normoxic” degradation is facilitated by the hydroxylation of conserved proline residues of the *α* polypeptides mediated by the prolyl-4-hydroxylase-1, prolyl-4-hydroxylase-2, and prolyl-4-hydroxylase-3 (PHD1, PHD2, and PHD3) [34]. Hydroxylation renders *α* subunits bound to the E3 ubiquitin ligase component von Hippel-Lindau (pVHL) protein leading to their proteasomal breakdown and absence of functional heterodimers in “normoxic” cells [33]. Under hypoxia, in contrast, PHDs become inactive, HIF-*α* subunits stabilize and dimerize with their *β* counterparts and transactivate adaptive target genes. These include not only genes of the glucose and lipid metabolism but also the ones encoding for regulators of proliferation, survival, DNA repair, cytoskeletal components, extracellular matrix-related proteins, cyto-, and chemokines [35]. Since BMSCs express both HIF-1 and HIF-2*α* and the HIF-orchestrated cellular hypoxic response is fully functional in these cells, one can speculate that differential activation of the underlying molecular system consequently affects the putative immunomodulatory nature of these cells too [36].

## 4. The Hypoxic Response of BMSCs

### 4.1. Metabolic Adaptation

One of the critical aspects of the HIF-governed hypoxic adaptation is the metabolic switch from the oxidative phosphorylation to less oxygen-dependent metabolism. Since in BMSCs both the aerobic glycolysis and oxidative phosphorylation are active and the HIF system is also intact and functional, one can speculate if the HIF-orchestrated metabolic switch remains active in the *ex vivo* expanded BMSC cultures [37]. *In vitro*, it, apparently, does since hBMSCs exposed to 2% oxygen show elevated glucose consumption compared to cells cultured under atmospheric oxygen conditions [38]. Parallel, the incorporation of glucose-derived carbons into citrate, which reflects the rate of the glycolysis-driven TCA cycle, is significantly reduced. Despite this reduction, however, citrate carbons are still mainly derived via pyruvate dehydrogenase indicating that basal activity of the TCA cycle remains intact even under oxygen-depleted conditions [39]. Interestingly, although this metabolic switch under hypoxia should also be reflected in lactate production, there are contradicting results in relation to lactate production of hypoxic BMSC cultures. While some studies observed decreased extracellular lactate levels in the culture media of oxygen-deprived BMSCs, others reported elevated lactate production under hypoxic conditions [37, 38, 40]. Recent systemic analyses of the hypoxic BMSC metabolome detailed the picture further showing that elevated extracellular lactate levels are accompanied by unchanged intracellular lactate levels suggesting the existence of a high-capacity lactate export system in BMSCs [39]. Since lactate export seems to become saturated upon *in vitro* osteogenic differentiation, one may wonder if a differential proportion of undifferentiated species in the BMSC cultures examined is accounted for the reported conflicting results in lactate production.

In glutamate metabolism, which serves as carbon and nitrogen supplies alike, hypoxic BMSCs display different kinetics as well. Under hypoxia, they show an increase in TCA cycle-driven metabolism of glutamate and this, in conjunction with the elevated glucose consumption, may be related to the activated malate-aspartate shuttle observed [39]. Data suggest that this metabolic profile allows hypoxic BMSCs to maximize their ATP production at reduced glycolytic carbon supply of the TCA cycle. In addition, increased glutamate metabolism in oxygen-deprived BMSCs is accompanied by reduced production of ammonia, the by-product of glutamate metabolism, suggesting that glutamate conversion is, primarily, mediated by transaminases instead of the ammonia-producing glutamate dehydrogenase in hypoxic BMSCs [38, 39]. Since the transaminase pathway of glutamate metabolism facilitates generation of nonessential amino acids, one can speculate that the increased glutamine consumption of hypoxic BMSCs mainly serves their translational machinery [41]. This is in accordance with the findings that oxygen-depleted BMSCs secrete a number of soluble factors with potential impact on the inflamed microenvironment and the hypoxic glutamine metabolism may serve the reprogrammed translation of hypoxic BMSCs. It is also noteworthy that normoxic cultures are reported to produce ammonia at concentrations that are believed to be inhibitory *in vitro* so one can speculate if the hypoxia-adapted glutamine metabolism with reduced ammonia production is reflected in the proliferative capacity of *ex vivo* expanded BMSC cultures [42].

### 4.2. Proliferation of Hypoxic BMSCs

Indeed, BMSC cultures expanded at oxygen levels lower than 3% are reported to show better proliferative capacity and consistently higher cumulative population doublings compared to cells kept under atmospheric oxygen conditions [36, 38, 43]. This may be critical for BMSC-based therapeutic applications since these modalities require *ex vivo* expansion of cells to be transplanted due to the low frequency of BMSCs in source marrow isolates [4]. Analyses of proliferation kinetics revealed that hypoxic cells enter the cell cycle faster and start *in vitro* cell division earlier than that of the normoxic ones [38]. Although details of the underlying mechanisms including the role of reduced production of ammonia are still not fully understood, a number of parallel events, which may potentially orchestrate the hypoxia-driven upregulation of BMSC proliferation, have already been reported.

**Figure 2:**
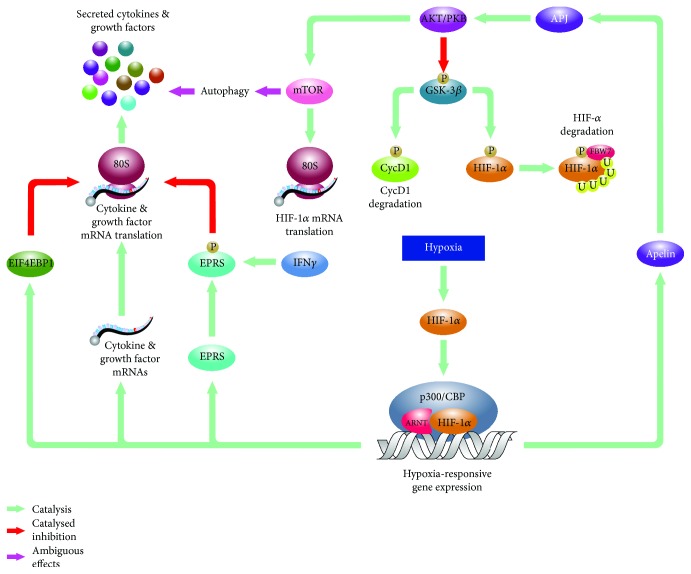
Molecular mechanisms involved in the proliferative and cytokine response of hypoxic BMSCs. Hypoxia-stabilized HIFs induce genes like *APLN* that, in return, activate the AKT/PKB pathway. This leads to inactivating phosphorylation of GSK3*β* releasing cyclin D1 from GSK3*β*-mediated inhibition. Data also suggest that the activation of the AKT/PKB results in the regulation of mTOR that affects both autophagic and translational activities of BMSCs. Besides mTOR, hypoxia also induces genes like *EPRS* and *IEF4EBP1* that also contribute to the hypoxia-specific translational pattern, likely, to define composition of secreted immune-modulatory factors of BMSCs.

One of these mechanisms is mediated by the APELIN-AKT/PKB axis in hypoxic BMSCs (Figure 2) [43]. APELIN is the endogenous ligand for the orphan G protein-coupled receptor APJ, and the APELIN-encoding *APLN* gene is induced in a HIF-1*α*-dependent manner in hypoxic BMSCs [44, 45]. *In vitro* studies using rodent BMSCs revealed that APELIN-mediated activation of APJ leads to the inactivating phosphorylation of glycogen synthase kinase 3 beta (GSK3*β*) via the AKT/PKB in a phosphoinositide 3-kinase- (PI3K-) dependent manner [43, 46]. One of the known targets of GSK3*β* is cyclin D1, the regulatory component of the cyclin D1/cyclin-dependent kinase 4 (CycD1/CDK4) complex that governs the G_1_/S phase transition in the cell cycle [47]. The GSK3*β*-mediated phosphorylation of cyclin D1 results in nuclear export and the cytoplasmic degradation of the latter one leading to inactivation of the CycD1/CDK4 complex. Thus, experimental data suggest that, in hypoxic BMSCs, the HIF-induced *APELIN* triggers the AKT/PKB axis that results in the inactivation of GSK3*β* and, consequently, upregulation of the CycD1/CDK4 complex and the G_1_/S phase transition [46]. Since in, cancer cells, AKT/PKB-mediated inactivating phosphorylation of GSK3*β* contributes to the cytoplasmic stabilization of HIF-1*α* as well, one can speculate if an APELIN-AKT/PKB-HIF-1*α* axis forms a feed-forward regulatory loop in hypoxic bone marrow-derived mesenchymal stromal cells [48]. Moreover, since the translational regulator mammalian target of rapamycin (mTOR) is also a known effector of AKT/PKB in established cellular models, it would be interesting to see how the hypoxia-upregulated AKT/PKB contributes to the altered ammonia production via, for instance, the mammalian target of rapamycin (mTOR) pathway in hypoxic BMSCs.

### 4.3. Cytokine Production of Hypoxic BMSCs

Hypoxia-stabilized HIFs target hundreds of genes mostly inducing their expressions. This leads to complex modification of the gene expression pattern of BMSCs as it has been shown using oxygen-depleted rBMSCs [49]. Target genes include those encoding for proteins with known or predicted secretory functions that may exert immune-modulatory effects [43, 50]. One of the potential mediators of these effects is the robustly hypoxia-induced macrophage migration inhibitory factor (*MIF*) that, although traditionally has been considered to be a proinflammatory cytokine, can function as a mediator of the monocyte/macrophage arrest as well by acting as a noncognate ligand for the chemokine receptors CXCR2 and CXCR4 [51]. Another candidate target is *PTGES* that encodes for the prostaglandin E synthetase, suggesting elevated PGE2 synthesis in hypoxic BMSCs. PGE2 has been reported to support monocyte differentiation into type 2 macrophages (M*ϕ*2) that are known activators of regulatory T lymphocytes (T_reg_) [52]. Since this raises the fact that BMSCs apply their immune-modulatory effects, at least in part, via the PGE2-M*ϕ*2-T_reg_ axis, it would be interesting to see if differential expression of the transforming growth factor beta (TGF*β*), which also promotes T_reg_ formation, exists in hypoxic BMSCs and if so, it contributes to the immune-modulatory properties of hypoxic BMSCs [53]. Apparently, this concept is underpinned by the findings that hypoxic mBMSCs trigger both proliferation and viability of the M*ϕ*2 fraction via a cell-to-cell contact mechanism that is, at least in part, mediated by M-CSF and ICAM-1 [54].

Whether hypoxia mediates similar alterations in the gene expression profile of human BMSCs and, if so, how these differentially regulated genes contribute to the observed immune-modulatory effects of BMSCs in inflammatory conditions need further investigations. However, not only *bona fide* secretory proteins may have a role in the immune-modulatory effects observed in relation to BMSCs. Indeed, hypoxia upregulates *EPRS* that encodes the glutamyl-prolyl-tRNA synthetase. Although it is primarily known as a cytoplasmic enzyme that catalyzes aminoacylation of glutamate and proline tRNA species, it also suppresses translation of diverse inflammatory mRNAs by binding their 3′-UTRs upon interferon-gamma-mediated phosphorylation [55]. Moreover, since proteolytic fragmentation of the tyrosyl-tRNA synthetase generates polypeptides that affect neutrophil chemotaxis by binding the CXCR1 chemokine receptor, one can speculate if hypoxic upregulation of the glutamyl-prolyl-tRNA synthetase in BMSCs has similar immune-modulatory functions [56].

The complex effects of hypoxia on the translational regulation of BMSCs are further indicated by the hypoxic induction of the eukaryotic initiation factor 4E-binding protein 1 (EIF4EBP1), a suppressor of 5′-CAP-dependent translation observed in rBMSCs [50, 57]. In established cell lines, oxygen depletion activates the AKT/PKB pathway that leads, among others, to the activation phosphorylation of the mTOR. As it has been discussed above, in mouse BMSCs (mBMSCs), the proximal section of the putative AKT/PKB-mTOR-EIF4EBP1 axis is activated by the hypoxia-inducible *APLN* [45]. Since mTOR is a known regulator of EIF4EBP1, one may wonder if the hypoxia-responsive, translational pattern-regulating AKT/PKB-mTOR-EIF4EBP1 axis exists in human BMSCs [50]. The finding that the hypoxia-mediated secretion of soluble factors like VEGF, FGF2, IGF-1, and HGF is sensitive to PI3K inhibitor 3-methyladenine (3-MA) in mBMSCs, apparently, supports the concept that a hypoxia-responsive AKT/PKB-mTOR-EIF4EBP1 pathway participates in the translation of cytokines/growth factors [58].

Interestingly, recently, it was also reported that siRNA-mediated knockdown of ATG7 attenuates the increased secretion of growth factors that suggests an interplay between the upregulation of growth factor secretion and ATG7-governed functions like, for instance, vacuole transport or autophagy [58]. Since autophagy, which is traditionally concerned as an mTOR-governed process, contributes to cell survival, the role of ATG7 in the cytokine secretion suggests a potential link between the immune-modulatory effects and viability of hypoxic BMSCs as well.

### 4.4. Hypoxia Affects Viability of BMSCs

One of the most profound effects of hypoxic exposure on BMSCs is shifted proliferation that raises the question if hypoxic exposure leads to premature senescence and, thus, exhausted immune-modulatory capacity of BMSC cultures. Apparently, some experimental data support this concern as far as the relative telomere length of hypoxic BMSCs was found shorter than that of the cells kept under atmospheric oxygen conditions [38]. In accordance, some studies reported an increased rate of apoptosis in BMSC cultures kept under hypoxia [59–61]. Still, it is widely believed that viability is preserved in *bona fide* hypoxic BMSC cultures as illustrated by the increased colony-forming unit values observed in hypoxic BMSC cultures [38, 62]. In accordance, hypoxia-stabilized HIF-1a has been shown to mediate the survival of rBMSCs in the presence of exosomes derived from oxidative stress neuronal cells [63]. One possible explanation for this controversy is that, in studies which reported elevated cell death, hypoxia was combined with serum deprivation so the observed apoptotic response may be accounted for the lack of vital nutrients rather than to low-oxygen levels. This notion is underpinned by the elevated glucose and glutamine consumption of hypoxic BMSCs discussed above. In terms of shortened telomeres reported in hypoxic BMSCs, data indicate that compensating prosurvival mechanisms may sustain viability of hypoxic cells. Indeed, both expression of LC-3, BECLIN-1, and ATG5, hallmarks of autophagy, and conversion of LC3B-I to LC3B-II, a marker of autophagosome formation, were reported in mBMSCs exposed to hypoxia [64]. The finding that induction of autophagy markers is sensitive to U0126, the selective inhibitor of the MAP kinases MEK1 and MEK2, indicates that hypoxia-triggered activation of autophagy is, at least in part, mediated by the MAPK pathway in mBMSCs [64]. The putative role of the MAPK pathway in the hypoxic response seems to be evolutionarily conserved as hypoxic activation of the ERK pathway has been shown in human BMSCs as well [36].

The potential importance of hypoxia-triggered autophagy may be illustrated by the observation that a short-term hypoxic exposure of mBMSCs protects cells from subsequent hypoxia/serum deprivation injury [58]. The protective effect of hypoxic preconditioning, in accordance with human models, is accompanied by increased levels of LC3 and BECLIN-1 further supporting the evolutionarily conserved aspect of the hypoxia-mediated upregulation of autophagy markers in BMSCs. Seemingly, induced autophagy makes mBMSCs more resistant to environmental stress. Indeed, hypoxia preconditioned mBSMCs are reported to show better survival after transplantation to infarcted hearts or when exposed to H_2_O_2_ [58, 65]. In support of this concept, HIF-1*α* overexpression, which may mimic hypoxic preconditioning, has also been shown to protect rBMSCs from oxygen-glucose deprivation-induced damage and this effect was correlating with the expression of autophagy markers [66]. Experimental data on non-preconditioned ischemic mBMSCs indicate that autophagy cannot rescue ischemic cells from apoptosis without mTOR activity and suggest that hypoxic preconditioning mediates resistance by upregulation of mTOR, probably, via the HIF-1*α*-APLN-AKT/PKB axis in the advancement of ischemic exposure [59]. Interestingly, shRNA-mediated knockdown of *ATG7* increased viability of hypoxic human BMSCs suggesting that, at least in hypoxic mBMSCs, ATG7 is not necessary to the hypoxia-responsive autophagy-mediated prosurvival mechanisms [67]. This observation also suggests that the role of ATG7 in hypoxia-responsive secretion of growth factors is more closely related to the vesicular transport functions of ATG7 than to its role in autophagosome formation.

This is in accordance with findings that indicate the importance of vesicular transport in BMSC-mediated immune-modulatory functions. Indeed, BMSC-derived exosomes have been reported to affect proliferation of cocultured cells and stem cell-derived exosomes have also been found to exert immune-modulatory effects [68, 69]. These data also question if live BMSCs are actually needed to reach the desired therapeutic effects in the cellular therapy of inflammatory conditions. Indeed, even ischemia-treated annexin V/propidium iodide-positive mBMSCs were shown to have immune-modulatory effects on cocultured macrophages [70]. The observed repression of inflammatory cytokines TNF-*α*, IFN-*γ*, IL-12, and IL-6 and induction of PGE2, VEGFA, angiopoietin 1 (Ang-1), keratinocyte growth factor (KGF), insulin-like growth factor 1 (IGF-1), platelet-derived growth factor B chain homodimers (PDGF-BB), and erythropoietin (EPO) in cocultured macrophages indicate that even damaged BMSCs could reprogram the cytokine/growth factor profile of surrounding phagocytes. The general perception of controversies between the lasting immune-modulatory effects and the short half-life of transplanted BMSCs together with the absence of recipient BMSCs in heart and lung transplants or the observations that intravenously administered BMSCs are mostly trapped in the lungs posttransplantation is, apparently, in accordance with the idea that BMSCs can exert their immune-modulatory effects, at least in part, indirectly [71–76]. In accordance, coculture experiments with damaged BMSCs suggest that immune-modulatory effects are, at least in part, accounted for phagocytotic capacity saturated by the cellular debris of transplanted BMSCs [69]. Interestingly, despite the fact that it is widely accepted that *ex vivo* culturing influences the phenotype and surface antigen pattern of BMSC cultures without making them immunogenic and that exosome-mediated horizontal transfer of the anti-inflammatory BMSC phenotype is an exciting potential mechanism for mediating the anti-inflammatory effects, little is known on the effects of the *ex vivo* expansion of BMSCs on their interplay with resident phagocytes posttransplantation [77, 78]. Accordingly, it would also be exciting to see if various *ex vivo* oxygen levels have any impact on the anti-inflammatory properties of BMSCs via, for instance, expression of neoantigen.

## 5. Conclusions

The discovery of multipotent species in adult tissues paved the way for the clinically efficient regenerative medicine. The idea that transplanted stem cells repair damaged tissues via their plasticity, however, has, slowly, been shifted to the concept that multipotent cells exert their biological effects indirectly. Apparently, this notion makes them particularly useful to treat inflammatory conditions, where soluble factors play pivotal roles. Still, clinical trials delivered perplexing results calling further investigations for understanding the mechanism of action of stem cell's immune-modulatory effects as well as for conditions that improve the efficacy of stem cell-based therapeutic modalities in inflammatory pathologies.

Indeed, over the past decades, BMSC-based cellular therapies have drawn great attention in the clinical practice. Indeed, BMSCs have been tried in a number of human pathologies that exert immune dysfunction or imbalance of the regulation of immune response where our current therapeutic repertoire is very limited. Still, despite promising preclinical data, clinical trials failed to deliver breakthrough results. A good example is graft-versus-host disease (GVHD) where BMSCs were used in a number of, mostly phase I and II, clinical trials for the treatment of both acute and chronic forms of GVHD. Unfortunately, while the use of BMSC-based cellular therapy in acute GVHD was reported advantageous by a number of reports, clinical trials found the same approach rather ineffective in patients who suffered from chronic GVHD [16, 79–83]. Multiple sclerosis (MS), which affects the central nervous system by demyelination of the motor axons, is another autoimmune pathology where no effective treatment is currently available. Progressive MS patients treated with BMSCs, however, showed partial responses, some degree of remyelination in affected CNS areas, and improved T_reg_ lymphocyte titers suggesting that cellular therapy may have genuine therapeutic potential in MS following improvement of its efficacy [84, 85]. Similar conclusions can be drawn from clinical trials targeting patients suffering from steroid-refractory systemic lupus erythematous, a potentially fatal multisystem autoimmune disease. These trials showed that BMSC infusions maintained patients in remission up to 18 months with elevated T_reg_ lymphocyte numbers [86] but simple repetition of BMSC transplantation did not improve the efficacy of the therapy [87].

Inflammation is always accompanied by hypoxia raising the question if differential oxygen levels throughout the therapeutic processes influence the immune-modulatory capacity of naturally hypoxic BMSCs. Indeed, data indicate that, in hypoxia, BMSCs are biasing their metabolic homeostasis toward aerobic glycolysis. This, combined with the observed glutamine-mediated anaplerosis, not only enables faster ATP generation in the absence of full-blown oxidative phosphorylation but also provides a range of metabolic intermediates that can fuel *de novo* synthesis of essential biomolecules, critical prerequisites of cell survival, translation, and secretory functions [88].

Considering their phenotypic analogy, it is not surprising that hypoxic BMSC metabolism resembles the one observed in cancer cells that also often show an extremely high rate of glutamine consumption and dependency [89]. Today, it is also widely accepted that inflammation is tightly linked to tumour formation and recent advances in immunotherapy of neoplasms substantiate the notion that tumour cells exert immune-modulatory properties. In addition, the striking variation in the immune profiles of distinct tumours suggests that transformed cells apply multiple mechanisms to attenuate immune reactions [90]. Indeed, cancer cells are reported to be able to recruit anti-inflammatory cells like T_reg_ lymphocytes and myeloid-derived suppressor cells or secrete soluble immunosuppressive factors like TGF*β*, IL-10, or PD-L1 [91]. Since BMSCs are expected to exert their immune-modulatory functions in pathologies with similarly diverse inflammatory backgrounds, it seems to be a fascinating question if any of the immune-modulatory mechanisms of cancer cells apply to BMSCs.

Indeed, loss of function mutations of TP53, for instance, attenuates cytotoxic T cell invasion of breast cancers [92]. In a number of cancers, the absence of physiologic TP53 functions activate the nuclear factor kappa B (NF-*κ*B) pathway, that is, typically, accompanied by the paralysis of immune cell influx of tumour mass [93]. Since some data suggest that TP53 may be repressed in hypoxic BMSCs as well, one may wonder if the NF-*κ*B pathway, the critical mediator of inflammatory responses, is upregulated in hypoxic BMSCs modulating their cytokine/chemokine production [61]. Since the activity of the NF-*κ*B pathway in hypoxic cells, apparently, depends on the cytokine profile of the extracellular milieu, one can hypothesize that the putative TP53-mediated, hypoxia-responsive activation of NF-*κ*B contributes to the cytokine/chemokine production of hypoxic BMSCs. In return, this may downregulate the same in neighboring hypoxic immune cells modulating their cytokine/chemokine profile and, thus, reactivity [94].

**Figure 3:**
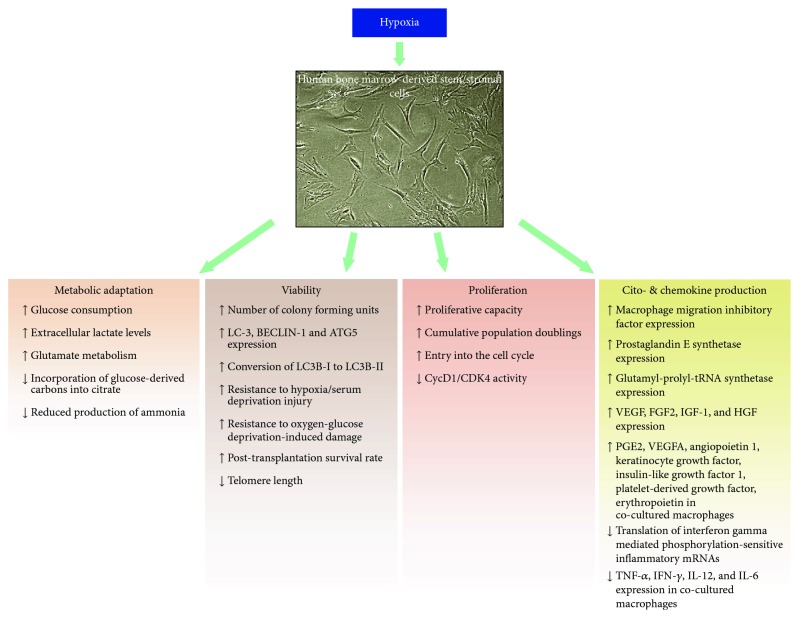
Summary of the key effects of hypoxia on BMSCs. The micrograph depicts human bone marrow-derived mesenchymal stem cells cultured under 2% oxygen in the absence of fibroblast growth factor-2 taken by the author using phase-contrast microscopy at 40x magnification.

Current data support the idea that hypoxic exposure of BMSCs pretransplantation may be one of the measures that improve their immune-modulatory effects posttransplantation (Figure 3). Data, however, also indicate that the prerequisite of an optimal hypoxic preconditioning protocol is the appropriate supply of nutrients like glucose and glutamine in order to fuel the hypoxia-reprogrammed translation of BMSCs with necessary metabolites. Nevertheless, the careful selection of supplements is underlined by the observation that ascorbic acid (AA) promotes BMSC proliferation [95]. Though the primary underlying mechanism is not clear due to its promiscuous metabolic roles, data indicate that exogenous AA mimics the effects of extracellular collagen fibers via increased collagen production, affects metabolism, and alters DNA methylation in BMSCs. Since AA, among others, acts as one of the cofactors of PHDs, one can speculate that AA might counteract the HIF-mediated mechanisms. Indeed, ascorbic acid, apparently, overrides the transcriptional activity of HIF triggered by deferoxamine (DFO), a routinely used hypoxia mimetic that, as an iron chelator, blocks the iron-dependent PHDs and therefore stabilizes HIFs. Since HIF transcriptional activity seems to be critical in the unfolding of the hypoxic BMSC phenotype, these observations illustrate that differential *ex vivo* culture conditions may provoke fundamentally different molecular mechanisms even in the presence of apparently equivalent macroscopic phenotypes.

These findings underline the importance of further optimization of the treatment regimens including manufacturing standards for future BMSC products. Experimental data not only indicate that activation of the molecular hypoxia-adaptive machinery can significantly contribute to the efficacy of BMSCs in inflammatory pathologies but also underline the importance of further research on the optimal *ex vivo* conditions, including hypoxia, for establishing enhanced anti-inflammatory BMSCs. Indeed, careful selection of the oxygen levels during isolation, *ex vivo* culturing, and posttransplantation seems to be one of the key aspects we need to consider in order to improve the efficacy of the clinical use of BMSCs. Hopefully, future *in vivo* studies focusing on the role of oxygen in BMSC-based cellular therapies of inflammatory conditions will answer this question.

## Figures and Tables

**Table 1:** Oxygen concentrations of various tissues.

Tissue/organ	O_2_ (%)	Reference
(i) Lung parenchyma (ii) Circulation (iii) Well-irrigated parenchymal organs	4-14	[96–104]
(i) Brain tissue	0.5-7	[105–108]
(i) Retina (ii) Corpus vitreum	1.0-5	[109, 110]
(i) Bone marrow	0-4	[1, 111]
